# Real-Time Direction Judgment System for Dual-Frequency Laser Interferometer

**DOI:** 10.3390/s24072030

**Published:** 2024-03-22

**Authors:** Qilin Zeng, Wenwei Chen, Hua Du, Wentao Zhang, Xianming Xiong, Zhengyi Zhao, Fangjun Zhou, Xin Guo, Le Xu

**Affiliations:** 1 College of Electronic Engineering and Automation, Guilin University of Electronic Technology, Guilin 541000, China; qilinzeng@guet.edu.cn (Q.Z.); hbchenwenwei@163.com (W.C.); duh@guet.edu.cn (H.D.); xmxiong@guet.edu.cn (X.X.); zhoufangjun@mails.guet.edu.cn (F.Z.); guoxin202103@163.com (X.G.); m18756615159_1@163.com (L.X.); 2 Key Laboratory of Optoelectronic Information Processing, Guilin University of Electronic Technology, Guilin 541000, China; 3 School of Precision Instrument and Opto-Electronics Engineering, Tianjin University, Tianjin 300072, China; 1023202035@tju.edu.cn

**Keywords:** direction judgment, FPGA implementation, laser interferometer, precision measurement

## Abstract

Current real-time direction judgment systems are inaccurate and insensitive, as well as limited by the sampling rate of analog-to-digital converters. To address this problem, we propose a dynamic real-time direction judgment system based on an integral dual-frequency laser interferometer and field-programmable gate array technology. The optoelectronic signals resulting from the introduction of a phase subdivision method based on the amplitude resolution of the laser interferometer when measuring displacement are analyzed. The proposed system integrates the optoelectronic signals to increase the accuracy of its direction judgments and ensures these direction judgments are made in real time by dynamically controlling the integration time. Several experiments were conducted to verify the performance of the proposed system. The results show that, compared with current real-time direction judgment systems, the proposed system makes accurate judgements during low-speed motions and can update directions within 0.125 cycles of the phase difference change at different speeds. Moreover, a sweep frequency experiment confirmed the system’s ability to effectively judge dynamic directions. The proposed system is capable of accurate and real-time directional judgment during low-speed movements of a table in motion.

## 1. Introduction

Ultraprecision displacement measurements of moving objects are a crucial research topic in many fields [1]. The tools used for ultraprecision displacement measurements include laser and grating interferometers [2,3,4]. Laser interferometers often use a phase subdivision method based on the amplitude resolution for high-precision displacement measurements. Optical signals are converted into sine and cosine electrical signals using an avalanche photon diode (APD). The resulting signals are then digitized using an analog-to-digital converter (ADC) and processed via a phase solution algorithm and subdivision process. The displacement result is obtained from the phase information [5,6,7,8,9,10].

This displacement measurement method is mainly influenced by the phase subdivision process and the ADC device when judging the direction of a moving table. The traditional method of direction judgment is primarily applicable when the phase subdivision is minimal and the ADC sampling rate is low. The direction of a table in motion is judged by the sign position of the sine–cosine electrical signal converted from the optical signal [11,12]. The sign bit of a positive cosine signal undergoes four relative changes: 00 → 10 → 11 → 01 → 00. Similarly, the sign bit of a negative cosine signal also undergoes four relative changes: 00 → 01 → 11 → 10 → 00. Here, 0 indicates that the sign bit of the positive cosine signal is negative, and 1 indicates that it is positive. This method is suitable for a phase subdivision of four. The update rate when changing the direction every quarter cycle does not affect the accuracy of the displacement measurement. With the advancement of electronic subdivision and the development of ADC devices, when the amplitude-based phase subdivision reaches 1024, the update rate of the traditional direction judgment method may not meet the requirement to be in real time. Additionally, the traditional method may result in a judgment error when the table in motion moves back and forth in a quarter cycle. The current direction judgment method, increasing the cosine signal change trend judgment compared to the traditional direction judgment method, greatly improves the judgment results of the real-time and quarter-cycle direction judgment errors [13]. When the amplitude-based phase subdivision reaches 4096 or higher and the ADC sampling rate reaches 160 Msps (million samples per second), the current method can result in good direction judgments above 100 kHz. However, for frequencies below 100 kHz, using the current method sacrifices the update rate of the direction judgment result to ensure its accuracy under the influence of signal noise. This is because it requires more clock cycles to detect the amplitude change in the low-frequency signal.

To obtain real-time and accurate low-frequency direction judgments, we analyze the signal characteristics of a dual-frequency laser interferometer and propose a real-time direction judgment system based on dynamic integration. Particularly, the movement direction is determined by the proposed system using an integration method. Real-time direction judgments are ensured by dynamically adjusting the integration time. At a low frequency of 100 kHz, the system accurately judges the direction of a table in motion in real time. The proposed system was implemented using field-programmable gate array (FPGA) technology and was applied to the phase solution platform of a dual-frequency laser interferometer.

## 2. Design of a Real-Time Direction Judgment System

**Figure 1:**
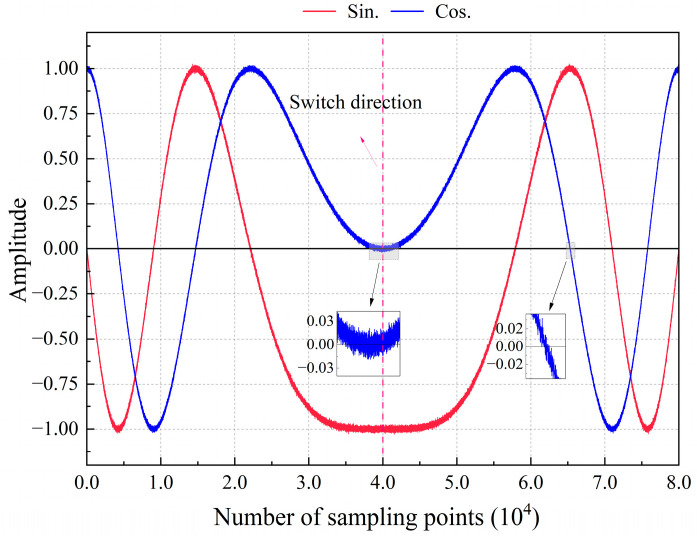
Schematic of a high-sampling-rate direction-switching signal.

Using an ADC with a higher sampling rate can improve the accuracy of displacement measurements by obtaining more complete photoelectric signal information and higher phase subdivisions. However, in terms of engineering, this will have some impacts on the direction judgment method, as shown in Figure 1. An ADC sampling rate that is too high means that the current direction judgment method needs more clock cycles for the signal amplitude change. Meanwhile, signal noise makes it difficult to distinguish the sign bit state of a signal when the direction changes and it crosses zero. Moreover, the actual working condition of the stationary state may not be an absolutely stationary state, and gradual drift may result in error accumulation.

As a result of these factors, the current method of making direction judgments is not suitable for research objects with a high sampling rate and high level of subdivision in motion at a low frequency.

**Figure 2:**
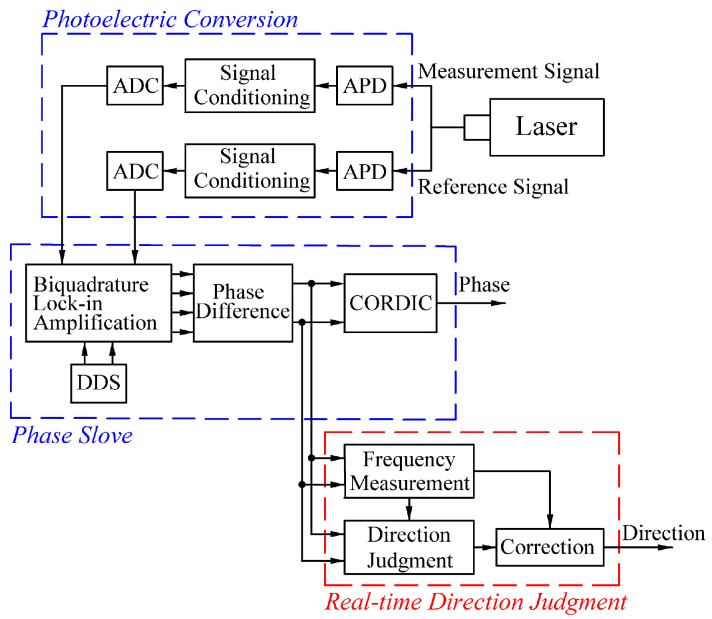
Block diagram of the proposed dual-frequency laser interferometer signal processing system.

To address the above problem, a dynamic real-time direction judgment system based on an integral dual-frequency laser interferometer was designed by analyzing the signal characteristics in a phase solution process (Figure 2). The laser signal is converted into an electrical signal by the APD in the photoelectric conversion section and conditioned. The conditioned signal is then converted into a digital signal by the ADC. The digital signal is subjected to biquadrature lock-in amplification and phase difference algorithms to eliminate errors [14,15,16]. Finally, the processed signal is subjected to the phase solution and real-time direction judgment processes to obtain phase and direction results.

### 2.1. Dual-Frequency Laser Interferometer Measurement Principle and Phase Solution Algorithm

**Figure 3:**
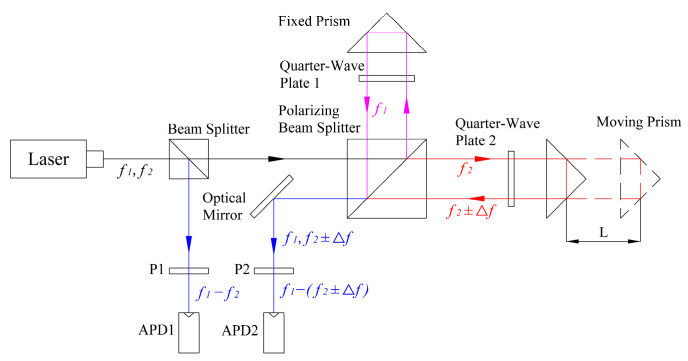
Schematic of the optical path of the dual-frequency laser interferometer.

The proposed system uses a dual-frequency orthogonal polarized laser with a vacuum wavelength of 633 nm and a frequency difference of 20 MHz ($f_{1}-f_{2}$). As shown in Figure 3, the laser beam emitted by the dual-frequency laser interferometer is split into two paths by a beam splitter. One path is used as the reference light and interferes after passing through polarizer 1 (P1), and a beat signal with a frequency of 20 MHz is synthesized. The other path is used as the measurement light, which, after passing through the measurement optical path, passes through polarizer 2 (P2) and then interferes, resulting in a beat frequency signal whose frequency contains a Doppler frequency shift.

The two beat signals are converted into electrical signals by the APD and then, after signal conditioning, are converted into digital signals by the ADC and input into the FPGA. The two signals can be expressed as follows:

(1) $$f_{r}=R\sin\left[2\pi \left(f_{1}-f_{2}+f^{\prime }\right)t\right]$$

(2) $$f_{m}=M\sin\left[2\pi \left(f_{1}-f_{2}+f^{\prime }+∆f\right)t\right]$$

where $f_{r}$ denotes the reference signal, $f_{m}$ denotes the measurement signal, $f_{1}-f_{2}$ denotes the dual-frequency laser frequency difference, f′ denotes the frequency uncertainty of the laser interferometer, ∆f denotes the Doppler frequency shift of the movement of the table in motion, and *R* and *M* denote the unequal amplitude errors of the two signals [17,18,19].

The Doppler frequency shift causes the phase difference between the reference and measurement signals to produce a periodic change in time T, which is expressed as T=1/2π∆f. T also denotes the time period of the current Doppler frequency.

To obtain quadrature signals with equal amplitudes that only contain the Doppler frequency shift ∆f, we demodulated the two signals in Equations (1) and (2) using biquadrature lock-in amplification. We opted for the more hardware-intensive digital method over the existing low-cost analog methods for this demodulation due to the following reasons:

1. Digital methods are more convenient when using signal processing algorithms to achieve a higher signal quality. For instance, in the case of a narrower bandwidth, the filtering effect of the digital method is stronger than that of the analog method. The filter is one of the crucial parts of signal demodulation in this study.

2. Phase solution boards have multiple optoelectronic signal acquisition channels. However, analog methods may increase the phase delay between channels due to differences in phase lock times or devices, and this must be avoided for the phase solution process.

3. Phase solution boards also have certain size requirements. If each channel is demodulated using analog methods, this will take up more space on the board, which is also a factor to consider.

The digital biquadrature lock-in amplification algorithm consists of two components: quadrature lock-in amplification and phase difference. The measurement signal and reference signal undergo quadrature lock-in amplification to produce two pairs of quadrature signals that do not contain the frequency difference $f_{1}-f_{2}$. These pairs are then subjected to the phase difference process to obtain the demodulated quadrature signals.

**Figure 4:**
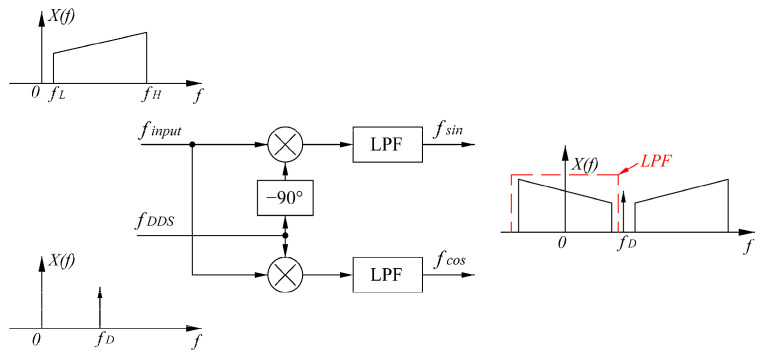
Block diagram of the quadrature lock-in amplification algorithm.

In this process, the two signals are mixed with a quadrature signal of frequency $f_{1}-f_{2}$ that is generated by direct digital synthesis (DDS). This mixing results in a spectrum shift of the signal, as shown in Figure 4. Both the frequency error generated by DDS and the frequency stability of the laser are negligible in subsequent calculations, as they are in the order of Hz. Therefore, they can be considered equal.

The spectrum-shifted four-channel signals are then passed through low-pass filters to generate two pairs of quadrature signals with only low-frequency content.

(3) $$f_{rsin}=\frac{1}{2}R\sin\left[2\pi \left(f^{\prime }\right)t\right]$$

(4) $$f_{rcos}=-\frac{1}{2}R\cos\left[2\pi \left(f^{\prime }\right)t\right]$$

(5) $$f_{msin}=\frac{1}{2}M\sin\left[2\pi \left(\;f^{\prime }+∆f\right)t\right]$$

(6) $$f_{mcos}=-\frac{1}{2}M\cos\left[2\pi \left(\;f^{\prime }+∆f\right)t\right]$$

The filtered signals are then passed through the phase difference algorithm to eliminate the laser interferometer frequency uncertainty $f^{\prime }$ in the signals. The sum-to-product identity formula is used, i.e., $\left(5\right)\times \left(4\right)-\left(6\right)\times \left(3\right)$ and $\left(6\right)\times \left(4\right)-\left(5\right)\times \left(3\right)$, to obtain the following:

(7) $$f_{sin}=\frac{1}{4}\mathrm{RM}\sin\left(2\pi ∆ft\right)$$

(8) $$f_{cos}=\frac{1}{4}\mathrm{RM}\cos\left(2\pi ∆ft\right)$$

Finally, a set of orthogonal signals with equal amplitudes and containing only the Doppler frequency shift ∆f is obtained. The arctangent calculation is then performed on this set (Equation (9)) to obtain the phase solution 2π∆ft. In this study, the coordinate rotation digital computer (CORDIC) algorithm was selected to perform the arctangent calculations on the FPGA [20,21,22,23,24]. The CORDIC algorithm has been extensively studied and will not be explained in detail in this article.

(9) $$2\pi ∆ft=\arctan\left(\frac{\frac{1}{4}\mathrm{RM}\sin\left(2\pi ∆ft\right)}{\frac{1}{4}\mathrm{RM}\cos\left(2\pi ∆ft\right)}\right)$$

Equation (10) describes the relationship between the level of movement L of the measured moving prism and the calculated phase based on the measurement principle and the structure of the laser interferometer shown in Figure 3. The wavelength of the laser is denoted by λ [25].

(10) $$L=\int_{0}^{t}\nu dt=\int_{0}^{t}\left(\frac{\lambda }{2}\times 2\pi ∆f\right)dt=\frac{\lambda }{2}\times 2\pi ∆ft.$$

The sign of the movement volume L represents the movement direction of the table in motion at one point in time, so the direction of the table in motion can be determined from the sign of the Doppler frequency shift. However, when the table in motion is operated at a low speed, the Doppler frequency shift is small. Judgments of the direction using this approach may be affected by signal noise. Therefore, we accurately judge the sign of 1/∆f and use real-time frequency measurement modules to ensure the direction judgments are made in real time.

### 2.2. Design of a Real-Time Direction Judgment System

**Figure 5:**
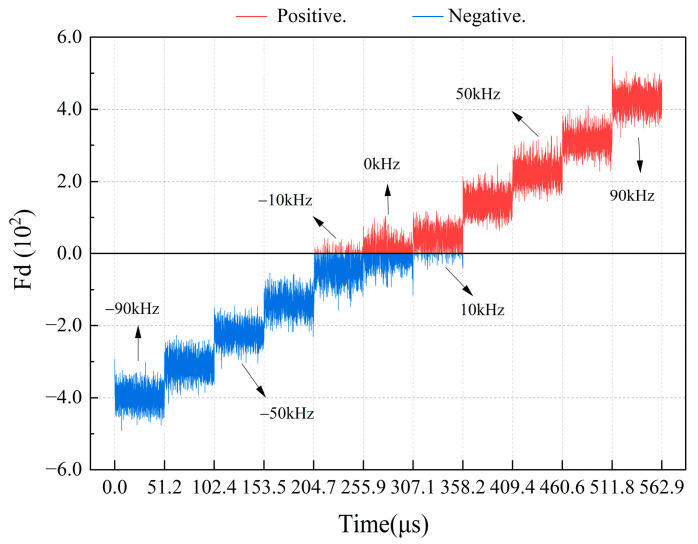
Schematic of direction judgment using the calculated positive and negative sign bits of Fd at low Doppler frequency.

For a high-speed and high-frequency table in motions, distinguishing the direction using the sign of the Doppler frequency shift yields highly accurate real-time results. However, in practical engineering, signal noise can induce inaccuracies in low-frequency states (Figure 5).

**Figure 6:**
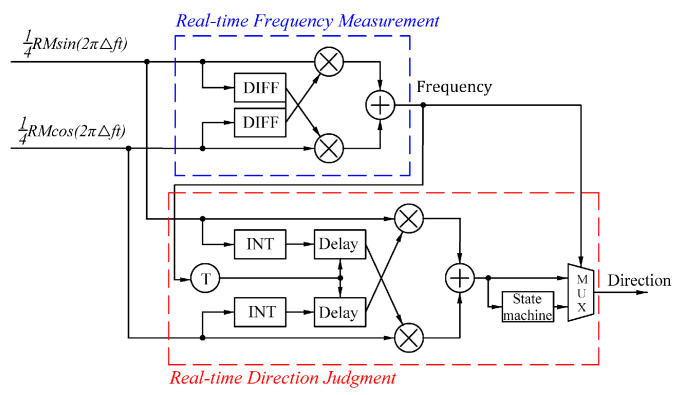
Block diagram of the real-time direction judgment of the system algorithm.

To ensure accurate directions during low-speed exercises and make real-time direction judgments during high-speed exercises, a real-time direction judgment system (Figure 6) was designed. The system includes a real-time frequency measurement module based on differentiation to calculate the Doppler frequency of the current signal. In addition, a real-time direction judgment module based on integration adjusts the integral time of the integral device to determine 1/∆f according to the current frequency to meet the system’s requirements for the accuracy of judgements in real time.

#### 2.2.1. Design of the Real-Time Frequency Measurement Module

The frequency of the Doppler effect changes when the table is in motion at different speeds. As the ADC sampling rate is fixed, signals of different frequencies will have a different number of sampling points per cycle. The real-time judgment module should ensure that the integral time of the integral device is appropriate for the current signal frequency, which requires it to rely on the update rate and a highly accurate real-time frequency measurement module.

To solve the aforementioned problems, a design based on a differential frequency measurement module was developed. The biquadrature lock-in amplification and phase differential algorithms produce an orthogonal signal used for differential calculations. The resulting output is as follows:

(11) $$f_{sin}^{\prime }=\frac{1}{2}\pi ∆fRM\cos\left(2\pi ∆ft\right)$$

(12) $$f_{cos}^{\prime }=-\frac{1}{2}\pi ∆fRM\sin\left(2\pi ∆ft\right).$$

By calculating $\left(7\right)\times \left(12\right)-\left(8\right)\times \left(11\right)$, we obtain the following:

(13) $$Fd=f_{sin}\times f_{cos}^{\prime }-f_{cos}\times f_{sin}^{\prime }=\frac{\pi ∆f{R^{2}M}^{2}}{8}.$$

**Figure 7:**
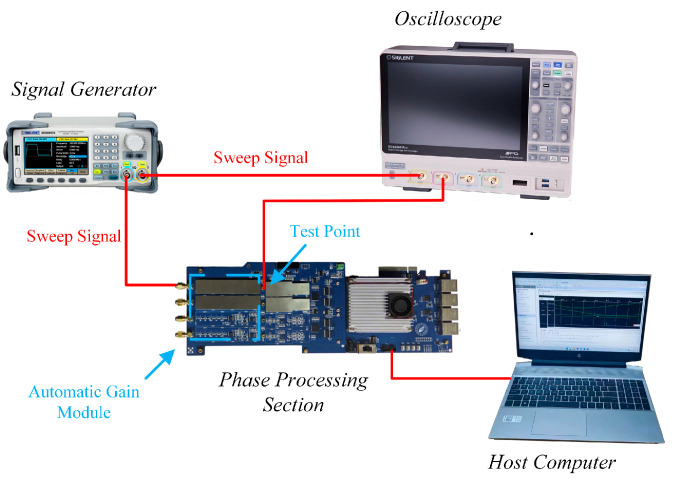
Schematic of the hardware-in-the-loop simulation experimental platform.

Equation (13) indicates that the frequency calculation result depends solely on the signal frequency and amplitude. The signal processing section includes an automatic gain compensation module that stabilizes the amplitude changes in the converted electrical signal within the same range under different working conditions [26,27]. Therefore, the amplitude’s effect on the linear relationship between Fd and the Doppler frequency is negligible. To verify this, we designed a hardware-in-the-loop simulation platform (Figure 7). This platform employs an SDG6052 signal generator to simulate the electrical signal after APD conversion, an SDS6000 Pro oscilloscope and host to obtain experimental data, and a signal processing section for the signal resolution.

The Kintex7-325T-2FFG900I FPGA chip produced by Xilinx Company in San Jose, CA, USA was utilized, as detailed in Table 1, which displays the number of resources. The resources most commonly used are lookup tables (LUTs), flip-flops (FFs), and multipliers (DSPs).

The maximum motion speed of the table in positive and negative motion in this study is 2.5 m/s, and the corresponding Doppler effect is frequently shifted to ±16 MHz. The frequency bandwidth of the input measurement light is 4–36 MHz after the photoelectric conversion because the $f_{1}-f_{2}$ of the dual-frequency laser is 20 MHz.

To verify the amplitude compensation effect of the automatic gain module, we performed frequency sweep tests with different amplitudes. We used a signal generator to produce sine frequency sweep signals ranging from 4 to 36 MHz with peak-to-peak values (VPP) of 500, 1000, and 1500 mV. We then used the oscilloscope to receive both the signal compensated by the automatic gain module and the signal directly output by the signal generator.

**Figure 8:**
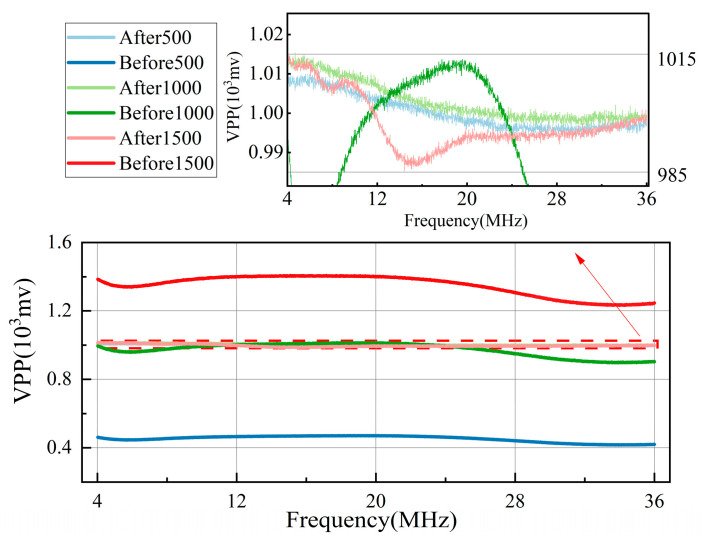
Experimental results of the amplitude compensation effect of the automatic gain module.

Figure 8 depicts the final experimental results. The data that underwent automatic gain compensation are labeled After500, After1000, and After1500, whereas the signals directly output by the signal generator are labeled Before500, Before1000, and Before1500. The figure shows that the peak-to-peak value of the signal after the gain compensation is between 985 and 1015 mV across the entire signal bandwidth, with a change of no more than 30 mV. The linear relationship between Fd and the Doppler frequency is not significantly affected by the amplitude.

**Figure 9:**
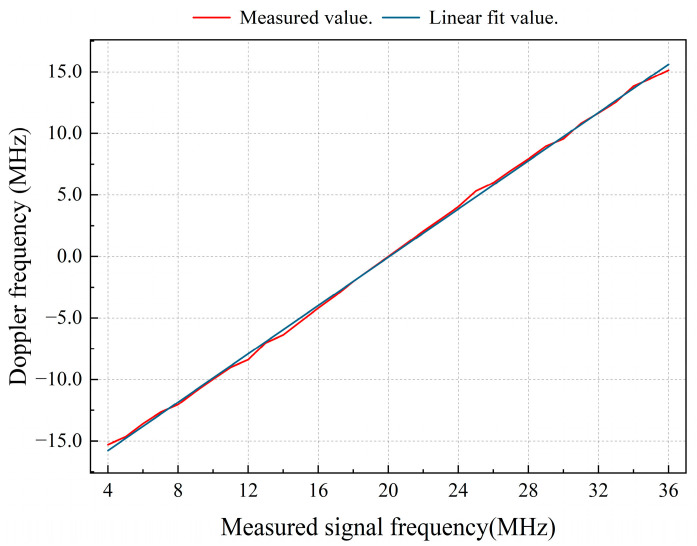
Relationship between the calculation results of the real-time frequency measurement module and the measured signal frequency.

To verify the relationship between the final frequency calculation result and the Doppler frequency, the actual frequency measurements were obtained for each frequency. A signal generator was used to produce the corresponding reference and measurement signals. In addition, the host continuously collected 8192 data points of actual frequency measurements from the FPGA probe for every 1 MHz in the range of 4–36 MHz. The collected data were used to calculate the average value and perform first-order linear fitting. Figure 9 shows the results. The calculation results of the real-time frequency measurement module have a linear relationship with the input frequency signals. Fd accurately reflects the Doppler frequency of the current signal.

#### 2.2.2. Design of a Real-Time Direction Judgment Module

**Figure 10:**
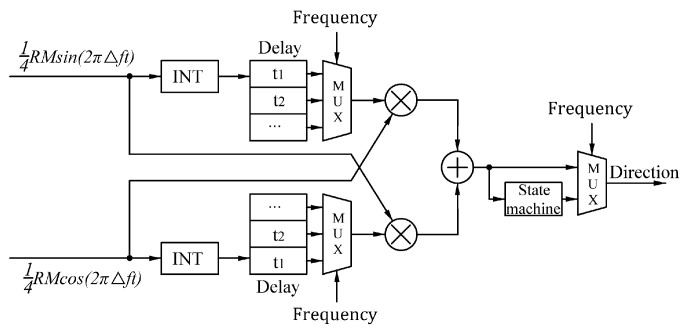
Diagram of the direction judgment module.

The real-time frequency measurement module allows for the selection of an appropriate integration time to calculate 1/∆f based on the frequency. However, incorrect integration time selection can result in direction errors. In addition, the integration time is limited due to the limited FPGA resources. To address these problems, a direction judgment module with a correction function for the output results is designed (Figure 10).

To calculate 1/∆f, we can theoretically perform an indefinite integration on the pair of orthogonal signals of Equations (7) and (8), as in Equations (14) and (15), respectively:

(14) $$\int \frac{1}{4}\mathrm{RM}\cos\left(2\pi ∆ft\right)dt\;=\frac{RM}{8\pi ∆f}RMsin2\pi ∆f$$

(15) $$\int \frac{1}{4}\mathrm{RM}\sin\left(2\pi ∆ft\right)dt\;=-\frac{RM}{8\pi ∆f}RMcos2\pi ∆f.$$

Equation (16) describes the expression for the final result, *Fi*, which is calculated by $\left(7\right)\times \left(14\right)-\left(8\right)\times \left(15\right)$. The movement direction of the table in motion can be determined by examining the sign of *Fi*.

(16) $$Fi=f_{sin}\times \int f_{cos}dt\;-f_{cos}\times \int f_{sin}dt\;=\frac{R^{2}M^{2}}{32\pi ∆f}.$$

However, in engineering applications, the signal is typically integrated using a definite integral, as shown in Equations (17) and (18).

(17) $$\int_{t_{1}}^{t_{2}}\frac{1}{4}\mathrm{RM}\cos\left(2\pi ∆ft\right)dt\;=\frac{RM}{8\pi ∆f}sin2\pi ∆ft{|}_{t_{1}}^{t_{2}}\;=\frac{RM}{8\pi ∆f}sin2\pi ∆ft_{2}-\frac{RM}{8\pi ∆f}sin2\pi ∆ft_{1}$$

(18) $$\int_{t_{1}}^{t_{2}}\frac{1}{4}\mathrm{RM}\sin\left(2\pi ∆ft\right)dt\;=-\frac{RM}{8\pi ∆f}cos2\pi ∆f{|}_{t_{1}}^{t_{2}}\;=-\frac{RM}{8\pi ∆f}cos2\pi ∆ft_{2}+\frac{RM}{8\pi ∆f}cos2\pi ∆ft_{1}$$

Equations (14) and (15) can be replaced by Equations (17) and (18), respectively, to perform the operation of Equation (16). The expression for the final result, *Fi*, of the definite integral can be obtained using $\left(7\right)\times \left(17\right)-\left(8\right)\times \left(18\right)$ as follows:

(19) $$Fi=\frac{R^{2}M^{2}}{32\pi ∆f}\left[1-cos2\pi ∆f\left(t_{2}-t_{1}\right)\right]$$

The table in motion’s fastest speed corresponds to a maximum Doppler frequency of 16 MHz with a period of 62.5 ns. If the integration time is less than 62.5 ns, errors may occur in the judgment of the direction or speed measurements due to signal noise, even though the calculated value of *Fi* is proportional to the Doppler frequency. If the integration time is longer than 62.5 ns, because it exceeds the time period of the maximum frequency, a proportional relationship between the calculated value of *Fi* and the Doppler frequency does not exist, so it is necessary to determine the appropriate integration time.

Equation (19) can be used to select the appropriate integration time. To avoid direction judgment errors caused by signal noise, it is crucial to ensure that the value of $cos2\pi ∆f\left(t_{2}-t_{1}\right)$) does not approach 1. For an input signal of a certain frequency, it is recommended that the integration time of the interval [$t_{1},t_{2}]$ does not approach integer multiples of period T, such as 0, T, and 2T. To the direction identification system operates in real time, the interval [$t_{1},t_{2}$] should be contained in [0,T] and should not approach the time points at either end.

**Figure 11:**
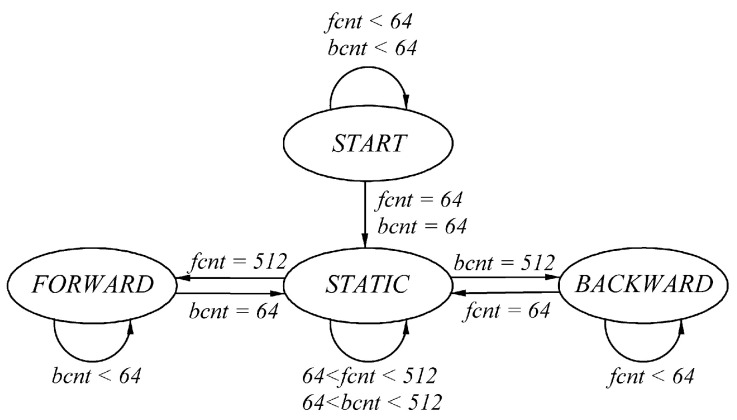
Schematic of low-frequency state machine’s state transition.

The 160 Msps, 12-bit, dual-channel, analog-to-digital ADS4226 converter chip is used for signal acquisition. The biquadrature lock-in amplification algorithm processes the acquired signals into quadrature signals with a Doppler shift range of ±16 MHz. In theory, it is necessary to judge the direction of the frequency range of DC-16 MHz; however, the stationary motion of the table does not necessarily mean absolute stillness, and slow drift will cause some frequency changes [28,29,30]. According to engineering requirements, if the Doppler frequency is below 300 Hz, which corresponds to a kinematic table velocity of approximately 0.22 mm/s, the table is considered stationary. For a Doppler frequency of 300 Hz, the number of sampling points in a single cycle is 530,000. Using only 1/∆f to determine the movement direction at 300 Hz would require a long integration time and consume an enormous amount of FPGA resources. To address this problem, a wide-in, tight-out state machine (Figure 11) was set up to ensure the accuracy of the low-frequency discrimination step while using as few FPGA resources as possible.

**Figure 12:**
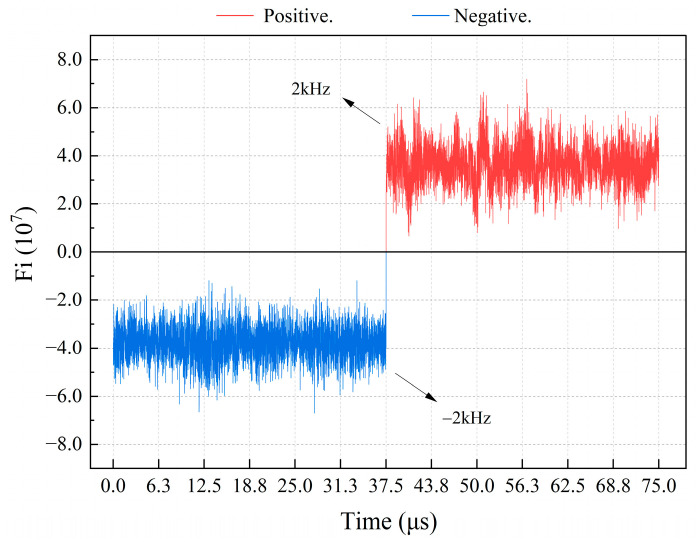
Calculation of Fi at a Doppler frequency of 2 kHz.

To simulate a Doppler frequency of ±2 kHz, the experimental device depicted in Figure 7 is employed to obtain the actual integration calculation result, *Fi*, using the host computer. By setting the integration time to 6400 ns, which is equivalent to 1024 sampling points, the direction of the current table in motion can be accurately determined from the sign of *Fi*. Figure 12 shows the results. In the actual process, since the Doppler frequency does not change suddenly, the integration time of 6400 ns is enough to make the correct direction judgment at a 2 kHz frequency, while maintaining the same update rate in the interval from 2 kHz to 20 kHz.

Based on the theoretical derivation of the direction judgment module, the real-time performance of the proposed direction judgment system must remain consistent across various Doppler frequency signals. According to the integration time required for real-time judgments when the Doppler frequency is 2 kHz, the entire Doppler signal bandwidth is divided into four intervals: 300 Hz–20 kHz, 20–200 kHz, 200 kHz–2 MHz, and 2–16 MHz, corresponding to integration times of 6400, 800, 50, and 6.25 ns, i.e., the sampling points are 1024, 128, 8, and 1, respectively. Considering the internal FPGA resources and technical requirements for identifying the direction of low-frequency signals with a Doppler frequency of 300–2000 Hz, we designed a wide-in, strict-out state machine. Figure 11 shows the state machine using continuous state counters fcnt and bcnt for forward and backward motion judgments. We start counting when the direction judgment results are continuously the same. The static state is entered only after 64 consecutive direction judgment results are obtained that are opposite to the current direction. In the static state, only 512 consecutive forward or backward results jump from the static state to the corresponding state. The start state is used as an initialization protection state to prevent the system from determining directions incorrectly when it is first started.

## 3. Experimental Results

**Figure 13:**
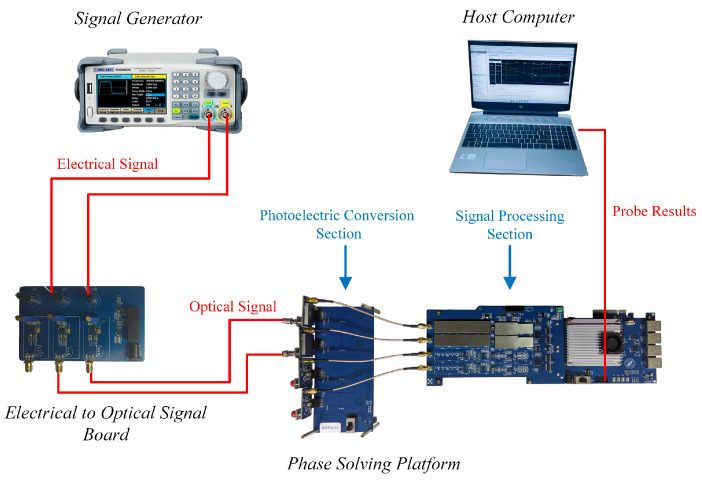
Schematic of the experimental device using simulated photoelectric signal solution.

To verify the effectiveness of the proposed real-time direction judgment system, we built a hardware-in-the-loop simulation platform (Figure 13). The SDG6052 signal generator outputs electrical signals, which are then converted into optical signals by an electrical-to-optical signal board to simulate the optical signals of the laser interferometer. The APD of the phase solution platform converts the optical signals into electrical signals for signal processing. We designed a circuit for the proposed real-time direction judgment system using the Verilog HDL language in the Vivado 2019.1 development environment and downloaded the circuit to the FPGA chip to conduct the algorithm test.

The system is minimally affected by the amplitude inequality of the quadrature signals and DC bias due to the hardware’s circuit auto-gain and the software’s dual-quadrature phase-locked amplification algorithms. However, the signal-to-noise ratio of the signals has a greater impact. To evaluate the system’s performance, the quadrature signal is acquired from the FPGA and its signal-to-noise ratio is calculated as shown in Table 2. The quadrature signal’s signal-to-noise ratio is approximately 30 dB across the entire frequency bandwidth at which the direction judgment is made.

**Figure 14:**
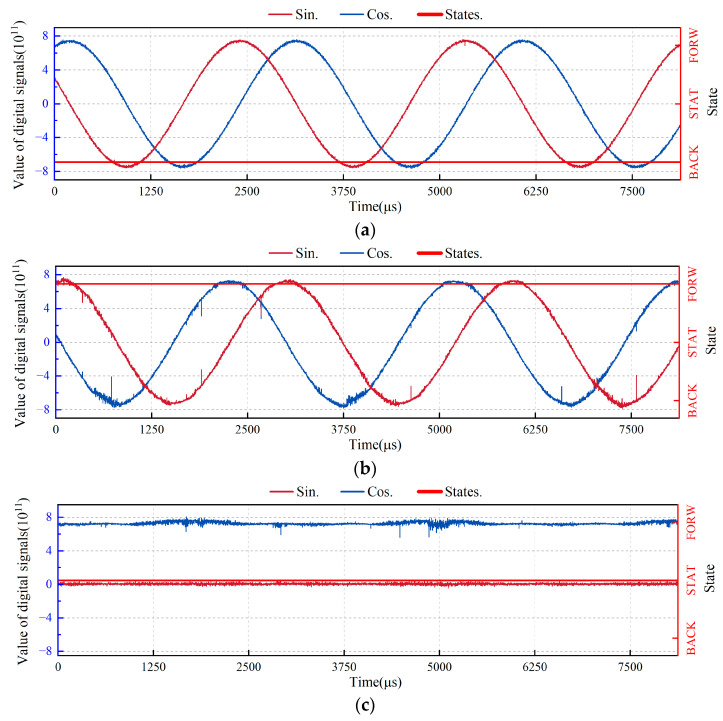
Results of low-frequency accuracy test of the real-time direction judgment system. (**a**) Direction judgment results when the Doppler frequency is −300 Hz; (**b**) Direction judgment results when the Doppler frequency is 300 Hz; (**c**) Direction judgment results when the Doppler frequency is zero.

To evaluate the accuracy of the proposed system in low-frequency motion states, we used the experimental device shown in Figure 13, which simulates the working conditions of −300, 0, and 300 Hz Doppler frequencies. The test results are displayed in Figure 14. The data collection method used was an integrated logic analyzer with 65,536 sampling points. The results show that the proposed system can accurately judge the direction of the table in motion when the Doppler frequency is ±300 Hz and can accurately judge that of the table in motion in a static state when the Doppler frequency is 0 Hz.

In Figure 14, the FPGA probe samples the quadrature signal every 125 ns, resulting in a total acquisition time of 8,192,000 ns (125 ns × 65,536). This acquires 2.7 cycles of the quadrature signal, corresponding to a Doppler frequency of approximately 300 Hz.

**Figure 15:**
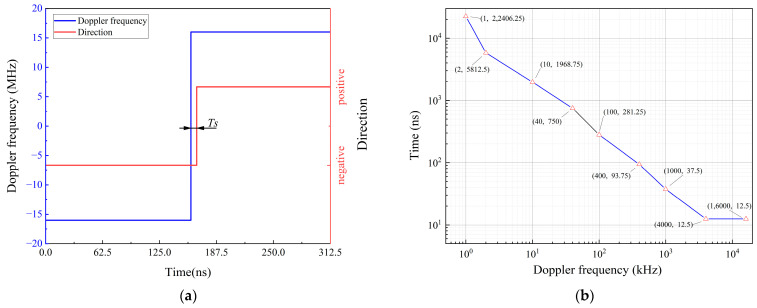
Experimental results of the real-time direction judgment system’s response speed: (**a**) 16 MHz frequency response diagram; (**b**) Other frequency response diagrams.

To evaluate the response speed of the proposed system in each frequency range, sudden changes in frequency were applied to the input signal. For example, as shown in Figure 15a, the Doppler frequency suddenly changes from –16 to 16 MHz, where Ts is the response time of the direction conversion. Figure 15b shows the experimental results in each frequency range. The results of the data analysis show that for the Doppler frequency in each range, the proposed system can make direction judgments within 0.125 T in the corresponding period.

**Figure 16:**
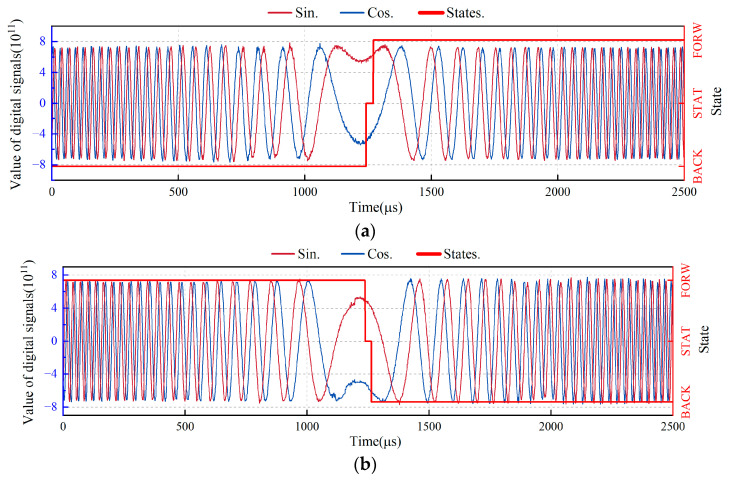
Experimental results of the accuracy of dynamic direction judgments using a real–time direction judgment system. (**a**) Direction judgment results when the Doppler frequency is changed from –100 to 100 kHz; (**b**) Direction judgment results when the Doppler frequency is changed from 100 to −100 kHz.

To evaluate the direction judgment results of the proposed system under dynamic conditions, a dynamic experiment was performed in which the table in motion moved from forward to static and then backward and from backward to static and then forward; the corresponding Doppler frequency change is −100 kHz → 0 kHz → 100 kHz and 100 kHz → 0 kHz → −100 kHz. Because the maximum acceleration of the table in motion was 32 m/s^2^ and the speed of the table in motion corresponding to the 100 kHz Doppler frequency was 0.075 m/s, the fastest time for the table in motion to complete a single round trip was 0.0046875 s. To simplify the frequency sweep signal input, we set the time to 0.005 s to complete the Doppler frequency sweep experiment of −100 kHz → 0 kHz → 100 kHz or 100 kHz → 0 kHz → −100 kHz. To better show that changes in sine and cosine lead to changes in direction, we captured a portion of the experimental results (Figure 16). Throughout the dynamic experiment, the proposed system had no direction misjudgment errors, which verified the dynamic characteristics of the system.

## 4. Conclusions

In this study, we proposed a dynamic real-time direction judgment system based on an integral dual-frequency laser interferometer to solve the problem of current real-time direction judgment systems, which are limited by the ADC sampling rate, resulting in inaccurate and insensitive direction judgments when the Doppler frequency is low. According to the current Doppler frequency, the integration time of an orthogonal signal is dynamically adjusted to ensure real-time direction judgments are made within the full Doppler frequency bandwidth. In addition, a state machine is set for the output directional result within a low-frequency bandwidth of 300 Hz–20 kHz, ensuring the minimal use of FPGA resources while meeting the system’s low-frequency judgment requirements. Through our experiments, we first demonstrated that the proposed system has good direction and static judgment capabilities for signals with Doppler frequencies of ±300 and 0 Hz, and then we showed that it can make direction judgments within 0.125 T in the period corresponding to the Doppler frequency of each range. Finally, the direction judgment accuracy of the proposed system under dynamic working conditions was verified.

## Figures and Tables

**Table 1:** Xilinx Kintex7-325T-2FFG900I chip resources.

Name	Number
LUT	326,080
FF	407,600
DSP	840
Block RAM (KB)	16,020
GTX Transceivers	16
GPIO	240

**Table 2:** Signal-to-noise ratio for quadrature signals.

Doppler Frequency	SNR (dB)
300 Hz	29.88
2 kHz	30.97
10 kHz	32.60
40 kHz	33.49
100 kHz	33.56
400 kHz	30.77
1 MHz	31.84
16 MHz	30.36

## Data Availability

The data presented in this study are available upon request from the corresponding author due to division of responsibilities.
